# Pancreatic Neuroendocrine Tumors: From Benchside to Surgical Treatment

**DOI:** 10.3390/medicina62030479

**Published:** 2026-03-03

**Authors:** Giovanni Conzo, Federico Maria Mongardini, Maddalena Paolicelli, Michele Klain, Giuseppe Bellastella, Alessandra Conzo, Zhou Bo, Eduardo Lanza, Leandra Piscopo, Renato Patrone

**Affiliations:** 1 Division of General, Oncological, Mini-Invasive and Obesity Surgery, University of Study of Campania “Luigi Vanvitelli”, 80138 Naples, Italy; giovanni.conzo@unicampania.it (G.C.); federicomaria.mongardini@unicampania.it (F.M.M.); aleconzo@hotmail.it (A.C.); eduardo.lanza@policliniconapoli.it (E.L.); 2 Department of Advanced Biomedical Sciences, University of Naples “Federico II”, 80138 Naples, Italy; michele.klain@unina.it; 3 Department of Advanced Medical and Surgical Sciences, University of Campania “Luigi Vanvitelli”, 80138 Naples, Italy; giuseppe.bellastella@unicampania.it; 4 Department of Hepatobiliary and Pancreatic Surgery, The Second Affiliated Hospital, Zhejiang University School of Medicine, Hangzhou 310003, China; 1509004@zju.edu.cn; 5 Radiology Department of Surgery, Medicine and Pharmacy, University of Sassari, 07100 Sassari, Italy; leandra.piscopo@gmail.com; 6 Hepatobiliary Surgical Oncology Unit, Istituto Nazionale Tumori IRCCS Fondazione Pascale-IRCCS di Napoli, 80131 Naples, Italy; renato.patrone@istitutotumori.na.it

**Keywords:** pancreatic neuroendocrine tumors, multidisciplinary management, mininvasive surgery, molecular targeted therapy, peptide receptor radionuclide therapy, quality of life

## Abstract

Pancreatic neuroendocrine tumors (pNETs) are rare, clinically heterogeneous neoplasms with rising incidence linked to improved diagnostics. This review examines pNET management, addressing epidemiology, classification, diagnosis, treatment, and emerging therapies. Epidemiologically, pNETs show higher prevalence in Western populations, with emerging associations to metabolic disorders. The 2022 WHO classification highlights distinct prognoses for well-differentiated NETs versus poorly differentiated NECs, guided by Ki-67 and mitotic indices. Non-functional tumors often present late, while functional variants manifest hormonal syndromes, necessitating tailored approaches. Advanced imaging (contrast-enhanced CT/MRI, ^68^Ga-DOTATATE PET) and endoscopic ultrasound-guided biopsy enable precise localization and grading. Surgical resection remains curative for localized disease, with minimally invasive techniques reducing morbidity. Active surveillance is favored for small (<2 cm), low-grade, non-functional tumors, while larger or aggressive lesions require resection. Systemic therapies, including mTOR inhibitors (everolimus), anti-angiogenics (surufatinib), and peptide receptor radionuclide therapy (PRRT), extend survival in advanced cases, though immunotherapy efficacy remains limited. Future strategies emphasize molecular profiling, biomarker development, and multidisciplinary integration to optimize outcomes. This evolving paradigm prioritizes precision medicine, balancing oncologic control with quality of life and functional preservation.

## 1. Introduction

Pancreatic neuroendocrine tumors (pNETs) are a rare and heterogeneous group of neoplasms arising from the endocrine pancreas. Although they represent only 2–5% of all pancreatic malignancies, their incidence has significantly increased in recent decades, primarily due to advancements in imaging techniques and diagnostic modalities [1]. pNETs exhibit a broad spectrum of biological behaviors, from slow-growing, indolent tumors to highly aggressive metastatic disease, posing significant challenges for clinical management.

Over the past two decades, improvements in diagnostic techniques and a deeper understanding of the genomic landscape have significantly contributed to the increased identification of non-functional pNETs [2]. Surgical management of pNETs requires comprehensive preoperative staging, including tumor localization, grading, functionality, hormonal assessment, and patient performance status. Multidisciplinary team evaluation is essential for these complex conditions [3]. According to current guidelines, parenchyma-sparing pancreatic resections represent the preferred approach for asymptomatic nonfunctional pNETs exceeding 2 cm in diameter and for all sporadic functional pNETs, except in cases with unresectable distant metastases [4]. Conversely, clinical and radiological surveillance is an appropriate option for non-functional pNETs smaller than 2 cm, as outlined by the Italian Medical Oncology Association’s guidelines for neuroendocrine neoplasms (NENs) [5].

Enucleation, distal pancreatectomy (with or without splenic preservation), central pancreatectomy, pancreaticoduodenectomy, and total pancreatectomy represent the available surgical options selected according to tumor characteristics and anatomical localization [6]. In recent years, the widespread adoption of minimally invasive surgical techniques has notably influenced clinical practice, with distal pancreatectomy increasingly favored over enucleation due to its technical feasibility and growing standardization [7].

The guiding question was: What Is the best available evidence to inform contemporary diagnostic and therapeutic strategies for pNETs, and where do relevant controversies and knowledge gaps remain?

This study aims to critically analyze recent advances in the multidisciplinary management of pNETs integrating epidemiological trends, innovations in diagnostic imaging and grading, minimally invasive surgical strategies, and targeted systemic therapies. The goal is to outline an updated framework for optimizing therapeutic decision making, balancing oncologic control, functional preservation, and quality of life, while addressing ongoing challenges in tumor classification and advancing precision medicine strategies.

This review provides a decision-focused synthesis integrating recent updates in classification, imaging, surgery, and systemic/radionuclide therapies.

## 2. Methods

This narrative review was conducted and reported in accordance with the Scale for the Assessment of Narrative Review Articles (SANRA) framework, with the aim of ensuring transparency, balanced evidence selection, and scientific rigor. The review addresses pancreatic neuroendocrine tumors (pNETs) across the clinical continuum, including classification and prognostication, diagnostic work-up, surgical management (with emphasis on minimally invasive and parenchyma-sparing approaches), and systemic/locoregional therapies.

### 2.1. Literature Research

A structured literature research was performed in PubMed/MEDLINE, Embase, Scopus, and the Cochrane Library for articles published from 1 January 2010 to 1 January 2025. To capture practice-defining recommendations and emerging evidence, we also conducted targeted searches of international guidelines, consensus statement and clinical trial registries to identify pivotal and ongoing/completed prospective studies relevant to pNET management. Reference lists of key publications and guidelines were manually screened to identify additional eligible sources.

Search strategies were adapted for each database using free-text terms and, where applicable, controlled vocabulary (MeSH/Emtree). Keywords were used in various combinations and iteratively expanded during the search, including: “pancreatic neuroendocrine tumor”, “pNET”, “neuroendocrine neoplasm”, “WHO 2022”, “grading”, “Ki-67”, “staging”, “CT”, “MRI”, “^68^Ga-DOTATATE PET”, “somatostatin receptor imaging”, “EUS”, “EUS-FNA”, “biomarkers”, “liquid biopsy”, “surgery”, “enucleation”, “parenchyma-sparing”, “distal pancreatectomy”, “pancreaticoduodenectomy”, “minimally invasive”, “laparoscopic”, “robotic”, “lymphadenectomy”, “active surveillance”, “somatostatin analogs”, “everolimus”, “sunitinib”, “temozolomide”, “capecitabine”, “PRRT”, “177Lu-DOTATATE”, “dosimetry”, and “immunotherapy”.

### 2.2. Study Selection and Eligibility

Authors identified as inclusion criteria for review: (1) English language studies including patients with pancreatic cancer; (2) Surgical treatment of duodenopancreatectomy for malignacy open or laparoscopic or robotic; (3) studies reporting on et least one intraoperative, post operative and long term oncological or clinical outcomes (operative time, intraoperative complication, estimated blood loss, blood transfusion rate, length of stay, R0 resection rate, lymph nodes retrieval, postoperative morbidity and mortality rate, disease free and overall survival rates, diabetes); (4) Surgery performed with curative intent. (5) Total pancreatectomy for cancer with or without spleen preservation.

Otherwise, exclusion criteria was: (1) Non-English studies; (2) Animal studies; (3) Abstracts, expert opinions, editorials and letter to the editors; (4) Studies reporting inadequate clinical data or less than one intraoperative, postoperative, and long term oncological or clinical outcomes (5) Studies including surgery for no malignancy reason (pancreatitis or other); (6) distal pancreatectomy with or without spleen preservation.

Two authors (M.P. and F.M.M.) independently screened titles/abstracts and assessed full texts for eligibility. Disagreements in selection or thematic categorization were resolved by consensus, with senior author (G.C. and R.P.) input when required.

## 3. Epidemiology

First described in 1869 as a distinct subset of NENs, pNETs are more prevalent in Caucasian populations (84%) and show a slightly higher incidence in males, with rates increasing with age [7]. pNETs represent a specific subgroup of gastroenteropancreatic neuroendocrine neoplasms (GEP-NENs) and are distinguished by their hormonal activity, clinical behavior, and treatment strategies. Their incidence has steadily increased over recent decades, largely due to advances in diagnostic imaging and improved disease recognition [8]. Dasari et al. [8] highlighted marked geographical variation in NEN epidemiology, reporting higher incidence rates in Western countries—approximately 6.98 cases per 100,000 annually in the United States—while lower rates have been historically observed in Asia and other regions. However, the global adoption of advanced diagnostic modalities is progressively reducing these disparities. A growing area of interest concerns the relationship between metabolic disorders and the development and progression of pNETs. Natalicchio et al. [9] emphasized a bidirectional association between conditions such as diabetes mellitus, obesity, and metabolic syndrome and GEP-NENs. These comorbidities may influence not only tumor development but also biological behavior and therapeutic responsiveness, underscoring the importance of integrated, multidisciplinary management. The ESMO Clinical Practice Guidelines [10] define current standards for the diagnosis, staging, and management of GEP-NENs, reinforcing the prognostic value of Ki-67–based grading and TNM staging. Similarly, ENETS Consensus Guidelines [11] refine stratification by distinguishing well-differentiated grade 3 pNETs from poorly differentiated neuroendocrine carcinomas (NECs), a crucial distinction given their markedly different clinical outcomes. More recent work by Zhang et al. [12] emphasizes how molecular and genomic tools are reshaping epidemiological understanding, although access to these technologies remains uneven across regions.

Overall, the evolving epidemiology of pNETs reflects a combination of improved diagnostic accuracy, better recognition of metabolic risk factors and growing integration of molecular profiling. Continued research is needed to address regional disparities and deepen the understanding of biological heterogeneity underlying these tumors [8].

## 4. Classification

According to the WHO 2022 classification, neuroendocrine neoplasms (NENs) are broadly divided into well-differentiated neuroendocrine tumors (NETs) and poorly differentiated neuroendocrine carcinomas (NECs), based on morphological features and proliferative activity assessed through mitotic count and the Ki-67 index [13]. Well-differentiated pNETs are graded as G1, G2, or G3, reflecting progressive increases in proliferative activity, while NECs are by definition high-grade tumors [14]. The diagnosis of pNETs relies on an integrated evaluation that combines histomorphology with immunohistochemical markers such as chromogranin A, synaptophysin, and Ki-67, which remains essential for grading [14]. Importantly, the updated WHO system emphasizes the need to clearly distinguish G3 NETs from NECs, as they differ substantially in biological behavior, prognosis, and recommended treatment strategies. Accordingly, pNET G3 is well-differentiated (organoid architecture/cytology preserved) with Ki-67 > 20% and/or >20 mitoses/2 mm^2^, whereas NEC is defined by poor differentiation (small-/large-cell morphology, marked atypia/necrosis) and typically shows aberrant p53 and/or RB1 loss supporting the diagnosis. Ki-67 alone is insufficient to label NEC; morphology is the primary discriminator [13,14,15].

International guidelines have further refined classification for clinical use. The European Neuroendocrine Tumor Society (ENETS) highlights the importance of multidisciplinary assessment and stresses the distinct management pathways required for functional versus non-functional pNETs [4]. Functional tumors secrete biologically active hormones that lead to specific syndromes, whereas non-functional tumors—representing approximately 90% of pNETs—remain clinically silent until advanced stages [16,17].

A minority of pNETs arise in the context of hereditary syndromes such as multiple endocrine neoplasia type 1 (MEN-1), Von Hippel–Lindau (VHL) disease, and neurofibromatosis type 1 (NF-1), although most cases occur sporadically [18]. Recognizing these hereditary conditions is clinically relevant, as they often present with multifocal disease and require dedicated surveillance strategies. To provide a more structured approach to diagnosis and management, the Japan Neuroendocrine Tumor Society (JNETS) developed a comprehensive framework that integrates imaging, pathology, and biomarkers into a unified diagnostic algorithm [19]. Similarly, the ESMO guidelines align with WHO and ENETS criteria, combining tumor grade, stage, and functional status to guide therapeutic decision-making [10]. Together, these classification systems emphasize a biologically centered view of pNETs, in which differentiation, proliferative activity, and functionality are central determinants of prognosis and treatment. The growing convergence among international guidelines reflects a shared effort to harmonize terminology and optimize patient stratification while acknowledging ongoing challenges in distinguishing high-grade NETs from NECs and integrating molecular profiling into routine clinical practice [11,12,13,20].

## 5. Clinical Presentation and Quality of Life

The clinical presentation of pNETs varies widely depending on functional status. Functioning tumors secrete bioactive hormones and typically present with well-defined endocrine syndromes, whereas non-functioning pNETs are often asymptomatic until they reach an advanced stage. In many cases, non-functioning tumors are detected incidentally or because of symptoms related to mass effect or metastatic spread [21,22,23].

Recent evidence suggests that age at onset may also influence presentation. Pulvirenti et al. [24] showed that early-onset pNETs often display distinct pathological features, including a higher prevalence of well-differentiated tumors and improved survival. Regardless of age, patients may experience non-specific symptoms such as abdominal pain, weight loss, nausea, or jaundice. In functioning tumors, clinical manifestations correspond to the hormone secreted: insulinomas may cause hypoglycemia, gastrinomas lead to peptic ulcer disease, and VIPomas typically induce severe diarrhea.

Quality of life (QoL) has become an essential component of pNETs management, as patients often experience chronic symptoms and prolonged disease courses. Neuroendocrine-specific modules, including the EORTC QLQ-GINET21 [25,26], have complemented general assessment tools such as the EORTC QLQ-C30. This tool captures symptoms unique to gastrointestinal and pancreatic NETs, such as hormone-related manifestations, gastrointestinal disturbances, and psychological burden.

Validation studies [25,27] underscore the relevance of these QoL instruments, demonstrating that even patients with relatively indolent disease may experience significant impairment across multiple functional domains. To address the limitations of broader NET-specific tools, the EORTC Quality of Life Group recently developed the PANNET module [28], designed specifically for pancreatic neuroendocrine tumors. This reflects the growing recognition of QoL as both a key clinical endpoint and a determinant of individualized management. A systematic review by Watson et al. [29] confirmed that patients with gastroenteropancreatic tumors frequently report reduced QoL across physical, emotional, and social domains, reinforcing the need for integrated psychosocial support and multidisciplinary care. Together, these findings highlight that clinical presentation and QoL are closely interrelated and that comprehensive patient assessment must extend beyond tumor biology to include functional impact and patient-reported outcomes.

## 6. Diagnosis of pNETs: Current Approaches and Perspectives

The diagnosis of pNETs requires a multimodal approach that integrates clinical evaluation, biochemical markers, advanced imaging, and cytopathological assessment, with tumor grading based on proliferative indices [30,31]. Serum biomarkers such as chromogranin A, pancreatic polypeptide, and neuron-specific enolase may support the diagnostic process, although their limited sensitivity and specificity prevent their use as standalone tools [30]. Imaging plays a central role in tumor detection, characterization, and staging. Contrast-enhanced computed tomography (CT) and magnetic resonance imaging (MRI) remain first-line modalities, with pNETs typically showing arterial-phase hyperenhancement [31]. Functional imaging—such as somatostatin receptor scintigraphy (SRS) and ^68^Ga-DOTATATE PET—has substantially improved the ability to identify well-differentiated tumors and detect metastatic spread [19,31]. These techniques complement conventional imaging, particularly in small lesions or in cases requiring detailed assessment of metastatic burden. Endoscopic ultrasound-guided fine-needle aspiration (EUS-FNA) has become indispensable in the diagnostic workflow. It enables precise localization of small lesions, assessment of local invasion, and acquisition of cytological specimens for histopathological evaluation [32]. Local invasion is also evaluated by EUS imaging rather than by FNAC, and is suggested by: (1) disruption of the pancreatic contour and loss of the hyperechoic interface with adjacent organs (duodenum/stomach), (2) vascular abutment/encasement (particularly >180° contact), vessel wall irregularity/stenosis or thrombosis (PV/SMV/SMA/CA), and (3) signs of ductal involvement (MPD or CBD narrowing/obstruction) and/or infiltration of peripancreatic fat.

EUS-guided tissue sampling plays a central role not only in tumor localization but also in preoperative grading of pancreatic neuroendocrine tumors. Several studies have demonstrated that Ki-67 assessment on EUS-FNA samples shows good overall concordance with surgical specimens, supporting its clinical utility in treatment planning. However, diagnostic accuracy may be limited by intratumoral heterogeneity and sampling variability, with a recognized risk of grade underestimation, particularly in intermediate- and high-grade tumors. These limitations underscore the importance of cautious interpretation of Ki-67 values obtained from small cytological samples and of integrating histopathological findings with imaging features and clinical behavior [33]. More recent evidence suggests that the use of EUS-guided fine-needle biopsy (FNB), providing histological core specimens, improves grading accuracy and Ki-67 reliability compared with cytology alone, especially when therapeutic decisions depend on precise tumor stratification. Nevertheless, even with FNB, discordance between biopsy and resection specimens persists, reinforcing the need for multidisciplinary discussion when biopsy-based grading is borderline or clinically discordant [34]. Numerous studies have demonstrated the feasibility and reproducibility of Ki-67 evaluation on FNA samples, although certain challenges—such as distinguishing grade 2 from grade 3 tumors—persist [35,36,37]. Inter-observer variability in Ki-67 interpretation remains a recognized limitation, reinforcing the need for standardized evaluation criteria [36]. Despite advances in imaging and cytopathology, several diagnostic pitfalls remain. Sampling errors, tumor heterogeneity, and overlap with other pancreatic neoplasms—including adenocarcinomas and solid-pseudopapillary neoplasms—often necessitate careful correlation of clinical, biochemical, radiological, and pathological findings [1,16]. Immunohistochemical confirmation using synaptophysin, chromogranin A, and Ki-67 remains essential for defining neuroendocrine differentiation and grading [16]. Emerging technologies offer promising additions to traditional diagnostic strategies. Liquid biopsy, circulating tumor DNA, and novel imaging tracers may enhance early detection and enable real-time monitoring of tumor biology [38]. In Japan, advances in diagnostic imaging and EUS-FNA techniques have contributed to earlier diagnosis and improved outcomes, reflecting the impact of high-quality diagnostic infrastructure [19]. Circulating biomarkers are also gaining importance; recent work has explored prognostic and predictive indicators for treatment selection, although challenges remain in detecting circulating tumor cells with currently available platforms. Overall, the diagnosis of pNETs relies on a comprehensive synthesis of clinical, biochemical, radiological and pathological data. While EUS-FNA and Ki-67 assessment remain cornerstones of modern diagnostic practice, future progress will depend on standardizing grading methodologies and integrating emerging molecular and functional tools to improve diagnostic accuracy and guide personalized treatment strategies.

EUS-guided ablative therapies (particularly EUS-RFA) are increasingly used in selected small, well-differentiated PanNETs—mainly in specialized tertiary centers—showing high pooled efficacy in a recent systematic review/meta-analysis (PMID: 36837560), but broader adoption is constrained by limited availability, need for expert operators, and still-incomplete long-term oncologic data [39].

## 7. Surgical Treatment of pNETs: An Updated Comprehensive Overview

### 7.1. The Management Strategy for pNETs

The management strategy for pNETs depends primarily on whether the tumor is functioning—producing a clinical syndrome due to hormone hypersecretion—or non-functioning, often detected incidentally or through symptoms related to mass effect [40,41,42]. Surgical resection remains the cornerstone of curative-intent treatment for both functioning and non-functioning tumors, with minimally invasive techniques, including laparoscopic and robotic approaches, playing an increasingly important role [20,43]. Functioning pNETs such as insulinomas, gastrinomas, and VIPomas generally require surgical removal regardless of size, given the morbidity associated with hormonal excess [6]. For small, benign insulinomas, parenchyma-sparing procedures like enucleation are preferred when malignancy is not suspected and the tumor is sufficiently distant from the main pancreatic duct [44]. Non-functioning pNETs present a more complex decision-making scenario. Surgery is typically indicated for tumors larger than 2 cm, those with a Ki-67 index above 3%, or when imaging suggests aggressive behavior [45,46,47]. Conversely, small (<2 cm), low-grade, non-functioning tumors without suspicious radiological features may be safely monitored with structured surveillance protocols [45,46,47]. The presence of synchronous liver metastases does not necessarily preclude surgery; selected patients may benefit from resection of both the primary tumor and metastases, either simultaneously or staged, with improved survival reported in appropriately chosen cases [48,49,50].

### 7.2. Surgical Techniques: Open, Laparoscopic, and Robotic Approaches

**Figure 1:**
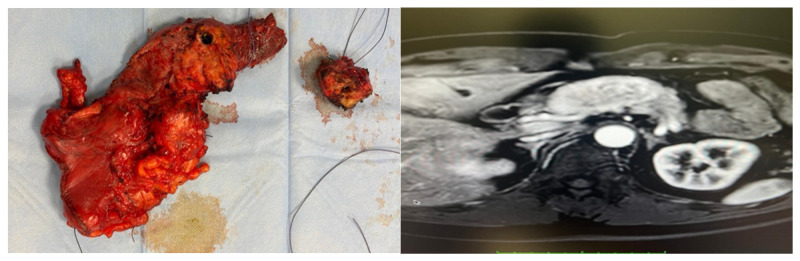
Imaging of a 51-year-old man demonstrating a 60 × 31 mm hypervascular solid mass at the pancreatic head–body junction, abutting the duodenum and splenoportal confluence. Central pancreatectomy with concomitant tail lesion enucleoresection confirmed PanNET and a low-grade IPMN (WHO 5th edition).

**Figure 2:**
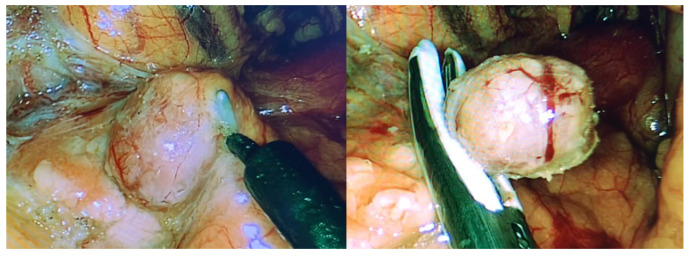
Laparoscopic enucleation for suspected insulinoma in a 32-year-old woman with documented endogenous hyperinsulinism (positive fasting test). Cross-sectional imaging and PET were negative; EUS identified a hypoechoic lesion in the pancreatic body. Frozen section was consistent with a pancreatic neuroendocrine tumor.

Open surgery historically represented the standard approach, particularly for complex cases involving vascular involvement, large tumors, or extensive nodal disease [49,51]. Procedures include pancreaticoduodenectomy for lesions in the pancreatic head and distal pancreatectomy for tumors of the body and tail, usually combined with lymphadenectomy (Figure 1). Minimally invasive surgery has significantly reshaped the surgical landscape. Laparoscopic pancreatic resections provide oncologic outcomes comparable to open surgery, with advantages such as reduced postoperative pain, shorter hospital stays, and faster recovery [50,51] (Figure 2). Laparoscopic or robotic distal pancreatectomy is now widely preferred for small, localized tumors of the pancreatic body and tail [52,53], while minimally invasive pancreaticoduodenectomy remains technically demanding and less commonly performed. Robotic-assisted surgery offers enhanced dexterity, three-dimensional visualization, and improved ergonomics [52,54,55]. Robotic distal pancreatectomy achieves similar oncologic outcomes to laparoscopy, with potentially lower conversion rates, although operative times and costs may be higher [54]. Robotic platforms are particularly useful for complex enucleations, especially when lesions are close to major vessels or the main pancreatic duct, and they facilitate organ-preserving procedures aimed to maintain endocrine and exocrine function [55,56].

### 7.3. Lymphadenectomy and Metastatic Disease Management

Lymph node status is a key prognostic factor in pNETs. Systematic lymphadenectomy is recommended for tumors larger than 2 cm, for pNETs with moderate to high proliferative activity, and when preoperative imaging suggests nodal involvement (Table 1 [48]). The role of surgery in metastatic disease—particularly in the presence of liver metastases—remains debated. Several studies support aggressive surgical management in selected patients, noting improved survival and symptom control [47,49,56]. Careful patient selection is crucial, with treatment decisions guided by tumor biology, disease distribution, and the likelihood of achieving complete or near-complete cytoreduction.

### 7.4. Global Perspectives on Surgical Management

International consensus documents, including those from the Chinese Study Group for Neuroendocrine Tumors [43] and the North American Neuroendocrine Tumor Society [40], emphasize the importance of individualized treatment strategies based on tumor grade, size, functionality, metastatic spread, and overall patient condition. Data from German [45] and Austrian [46] registries highlight variability in surgical approaches worldwide but consistently reinforce the central role of surgery in multidisciplinary pNET management. Robotic surgery continues to gain traction globally, supported by emerging evidence from high-volume centers demonstrating its safety, precision, and potential advantages in select cases [54,55]. Overall, surgical resection remains the fundamental treatment modality for pNETs and retains a key role even when limited metastatic disease is present. Advances in minimally invasive surgery have expanded operative possibilities, enabling safer and more refined procedures with improved perioperative outcomes [56]. Optimal management requires careful patient selection and integration of tumor biology, comorbidities, and surgical expertise. Continued prospective studies and international collaborations are needed to refine surgical indications and further improve long-term outcomes for this heterogeneous and challenging disease.

## 8. Alternative Treatment Protocols for pNETs: An Updated Overview

### 8.1. Targeted Therapies: mTOR Inhibitors and Anti-Angiogenic Agents

The mammalian target of rapamycin (mTOR) pathway is a key regulator of cellular proliferation in pNETs. Everolimus, the best-established mTOR inhibitor, has demonstrated meaningful antitumor activity across multiple trials. In the pivotal RADIANT-3 study, Yao et al. [57] reported a significant improvement in progression-free survival (PFS) compared with placebo, findings further supported by Thompson et al. [58] and Zitzmann et al. [59] in both clinical and preclinical settings. The RADIANT-4 trial extended these benefits to non-functioning neuroendocrine tumors of the gastrointestinal tract and lungs [60]. Additional evidence from the NECTOR trial [61] confirms everolimus activity even in patient’s refractory to platinum-based chemotherapy. Comparative analyses suggest that everolimus may retain an advantage over sunitinib in certain clinical contexts, although patient-specific factors remain influential [62]. Angiogenesis is a hallmark of neuroendocrine tumors, and its inhibition represents another effective therapeutic strategy. Surufatinib, a multi-target tyrosine kinase inhibitor with both anti-angiogenic and immunomodulatory properties, has emerged as a promising option. Xu et al. [63] demonstrated significant PFS improvement in the SANET-p Phase 3 trial, with subsequent analyses highlighting its potential place in treatment algorithms [64].

### 8.2. Peptide Receptor Radionuclide Therapy (PRRT)

PRRT with ^177^Lu-DOTATATE has become a major therapeutic advance for patients with progressive or advanced pNETs. The LUMEN study [65] and additional single-center experiences [66] confirm its safety and efficacy, particularly in well-differentiated gastroenteropancreatic NETs. These findings are further corroborated by recent single-center experiences and phase II registry studies, which demonstrate favorable efficacy and safety across diverse patient cohorts, including specific data from Japanese populations [67,68,69]. Combination protocols, such as the Lu-X regimen integrating metronomic capecitabine, have shown encouraging results in FDG-positive tumors, suggesting improved radiosensitization and disease control [67].

Harris and Zhernosekov [70], who underscore the growing relevance of individualized dosimetry and molecular profiling, have comprehensively reviewed the evolution of PRRT. This shift toward precision medicine is exemplified by the DUONEN trial, which utilizes dosimetry-guided protocols to balance maximum therapeutic intensity with the preservation of renal and bone marrow function [71]. PRRT can also be integrated with liver-directed therapies in patients with hepatic metastases, as discussed by Ngongoni and Visser [72]. Importantly, PRRT has demonstrated the potential to induce complete pathological responses and to enable conversion surgery in previously unresectable cases, as shown in reports by Umino et al. [73] and Sakaki et al. [74]. This expanding curative potential highlights the transition of PRRT from a purely palliative intervention to a key component of neoadjuvant-like strategies in the multidisciplinary management of pNETs.

### 8.3. Immunotherapy

Despite limited immunogenicity, immune checkpoint inhibitors (ICIs) have been explored in pNETs. The KEYNOTE-028 trial evaluated pembrolizumab in PD-L1-positive tumors, demonstrating modest but durable responses in a subset of patients [75]. Similar findings were reported in KEYNOTE-158 phase II study [76]. Beyond single-agent anti-PD-1 therapy, dual checkpoint blockade with ipilimumab and nivolumab has shown promising activity in advanced neuroendocrine tumors, as evidenced by a subgroup analysis of the CA209-538 trial [77]. Furthermore, the combination of sintilimab has demonstrated efficacy in previously treated metastatic neuroendocrine neoplasms [78], while the pairing of atezolizumab and bevacizumab—targeting both PD-L1 and VEGF—has provided clinical benefit, suggesting that combination strategies may overcome the low response rates of monotherapy [79]. As reviewed by Weber and Fottner [80], the success of ICIs in this setting likely requires biomarker-driven selection and a more refined understanding of tumor immune biology.

### 8.4. Somatostatin Analog (SSA)-Based Strategies

The therapeutic landscape for advanced GEP-NETs has transitioned from a standardized sequence of monotherapies to a multidimensional framework of precision oncology. While their role in symptom control for functional tumors is well-established, recent evidence suggests that the antiproliferative potential of SSAs like lanreotide is more biologically complex than previously understood. Mechanistic studies by Ungefroren et al. [81] have identified a critical crosstalk between different SSAs and TGF-β signaling pathways, suggesting that octreotide and lanreotide may exert divergent effects on neuroendocrine differentiation and cellular proliferation. These molecular insights provide a biological rationale for the clinical success of high-dose lanreotide regimens in advanced disease [82] and underscore the necessity of head-to-head comparisons to refine agent selection based on individual tumor biology [83].

This shift toward biological tailoring is further reflected in the emergence of synergistic combination strategies designed to overcome the limitations of SSA monotherapy in progressive disease. The SONNET study [84] has recently validated the integration of lanreotide Autogel with temozolomide, offering a robust therapeutic alternative for patients with progressive pancreatic and intestinal NETs by combining cytostatic and cytotoxic mechanisms. Meanwhile, the exploration of the immune microenvironment has led to the PLANET study [85], which investigated the safety and efficacy of pembrolizumab in combination with lanreotide depot. By pairing PD-1 inhibition with standard SSA therapy, this approach seeks to sensitize advanced, progressive GEP-NETs to immunotherapy, potentially redefining the management of tumors that have historically shown limited response to single-agent checkpoint inhibitors.

Data from Syguła et al. [86] have challenged long-standing clinical habits, indicating that in well-differentiated NETs where stable disease has been achieved via ^177^Lu-DOTATATE, the continued administration of SSAs may not offer significant incremental benefit. This highlights the need for a more evidence-based approach to treatment de-escalation and maintenance. Furthermore, the accurate assessment of response in this context remains a challenge; traditional RECIST criteria often fail to capture the nuanced metabolic and structural changes in Grade 1 and 2 tumors. To address this gap, the RECIN framework has been proposed as a specialized post hoc analysis tool for evaluating response following PRRT [87]. By integrating these novel evaluation frameworks with emerging data on molecular signaling and dual-agent combinations, clinicians can move toward a truly personalized treatment pathway that optimizes sequencing and maximizes long-term disease control in the modern era of GEP-NET management.

## 9. Discussion

pNETs represent a heterogeneous disease group whose rising incidence is largely attributable to improved diagnostic techniques rather than a true biological increase [8,79]. Initially described in 1869, they remain more common in Caucasian populations and slightly more frequent in males, with incidence increasing with age [7,20]. Apparent geographical differences largely reflect variability in healthcare systems and diagnostic resources rather than true epidemiological divergence [17]. Advances in imaging and classification systems have reshaped understanding of pNETs, yet several areas of uncertainty persist. One involves the relationship between metabolic disorders—such as diabetes, obesity, and metabolic syndrome—and tumor development or progression. Although a bidirectional association has been suggested, its causality and clinical implications remain unclear [8,9]. A major diagnostic challenge concerns distinguishing well-differentiated grade 3 tumors from poorly differentiated carcinomas, a distinction that carries significant prognostic and therapeutic implications [14,15]. Variability in Ki-67 assessment further complicates this process, highlighting the need for standardized pathology practices. International guidelines propose structured diagnostic algorithms that integrate imaging, pathology, and biomarkers. Insights from Japan [19] complement European and North American recommendations and reinforce the importance of harmonizing diagnostic strategies across different healthcare systems. Meanwhile, QoL assessment has become increasingly relevant. Tools such as the QLQ-C30, QLQ-GINET21, and, more recently, the PANNET module help capture the multifaceted burden experienced by these patients [25,26,27,28,29]. These instruments demonstrate that QoL may be significantly affected even in patients with seemingly indolent disease, underscoring the need for a patient-centered approach. Imaging remains a cornerstone of diagnosis and staging. While contrast-enhanced CT and MRI are widely used due to availability and accuracy for staging, functional imaging such as somatostatin receptor scintigraphy and ^68^Ga-labeled PET provides superior sensitivity for well-differentiated tumors and enhances metastatic detection [30,31,32,33,34,35,36,37,38]. Optimal diagnostic performance is achieved when conventional and functional imaging are used in a complementary fashion. Endoscopic ultrasound-guided fine needle aspiration (EUS-FNA) is essential for detecting small lesions and providing material for grading. Its role in Ki-67 assessment is well supported, though interobserver variability persists [33,34,35,36]. Despite progress, EUS-FNA still faces limitations related to sampling error and tumor heterogeneity, reinforcing the need for correlation with imaging and clinical data. Biomarkers continue to evolve, with emerging interest in circulating tumor DNA, circulating tumor cells, and other liquid biopsy strategies. Although methodological challenges remain, these approaches may improve early detection, risk stratification, and treatment monitoring [39,40]. Surgical resection remains the preferred curative strategy for localized disease and for functioning tumors due to the morbidity associated with hormonal syndromes [41,42,43]. Minimally invasive approaches offer comparable oncological outcomes with improved perioperative recovery, though surgical complexity and institutional expertise remain key determinants of success [20,50,51,52,53,54,55,56]. Management of non-functioning tumors smaller than 2 cm remains an area of debate: while size alone is not always predictive of aggressiveness, overtreatment and undertreatment both pose risks [42,45,46,47,48,49]. Decisions must therefore incorporate imaging features, proliferative index, and patient-specific factors. The introduction of robotic platforms has broadened operative options, particularly for organ-preserving resections and complex enucleations, though cost and availability limit widespread adoption [52,54,55]. Lymphadenectomy continues to hold prognostic significance, and its extent should be individualized based on tumor biology and imaging findings [48]. Surgery in the setting of metastatic disease remains controversial, with potential benefit restricted to carefully selected patients where complete or near-complete cytoreduction is achievable [45,46,47,48,49].

The management of pancreatic neuroendocrine tumors (pNETs) is rapidly evolving from a standardized sequence of treatments toward a sophisticated, multidimensional framework of precision oncology. Central to this shift is the refinement of Peptide Receptor Radionuclide Therapy (PRRT). While early data from diverse cohorts—including Japanese and Western populations—confirmed the general safety and efficacy of ^177^Lu-DOTATATE [67,68,69], the field is now pivoting toward individualized care. Nevertheless, as Harris and Zhernosekov [70] emphasize, the true future of PRRT lies in molecular profiling rather than “one-size-fits-all” dosing. This transition is best exemplified by the DUONEN trial, which utilizes dosimetry-guided protocols to maximize therapeutic intensity while meticulously safeguarding renal and bone marrow function [71]. The role of PRRT is also expanding from palliation to a “neoadjuvant-like” tool. Conversely to traditional views, systemic therapy is now a viable bridge to curative intent; studies by Umino et al. [73] and Sakaki et al. [74] have demonstrated that PRRT can induce pathological complete responses, enabling conversion surgery in previously unresectable cases. This multidisciplinary approach is further enhanced when integrated with liver-directed therapies for hepatic metastases [72]. mTOR inhibitors have demonstrated meaningful improvements in disease control [57,58,59,60,61], and their relative effectiveness compared with other targeted agents is increasingly studied [62]. Anti-angiogenic therapies, including newer agents with immunomodulatory effects, are expanding therapeutic possibilities [63,64]. PRRT has emerged as a major advance, with evidence supporting its safety, efficacy, and potential role in enabling conversion surgery in select patients [65,66,67,68,69,70,71,72,73].

The role of SSAs is also being re-evaluated through the lens of biological crosstalk. Furthermore, mechanistic studies indicate that octreotide and lanreotide may exert divergent effects on neuroendocrine differentiation through TGF-β signaling [81], necessitating a more tailored selection of agents [83]. While SSAs were once viewed purely as cytostatic, they are now being utilized as synergistic backbones. The SONNET study has validated the integration of lanreotide with temozolomide to combine cytostatic and cytotoxic mechanisms [84], while the PLANET study [85] investigated pairing lanreotide with pembrolizumab to sensitize tumors to immunotherapy.

A parallel debate exists within the realm of immunotherapy. Initial results from the KEYNOTE-028 and KEYNOTE-158 trials were somewhat underwhelming, showing that single-agent pembrolizumab yields only modest, albeit durable, responses [75,76]. Nevertheless, combination strategies are beginning to overcome the low immunogenicity of pNETs. Dual checkpoint blockade with ipilimumab and nivolumab [77] and the use of sintilimab [78] have shown significantly more promise. Furthermore, the pairing of atezolizumab and bevacizumab—targeting both PD-L1 and VEGF—suggests that synergistic inhibition is the key to clinical benefit in refractory patients [79].

Surgical resection remains the only potentially curative treatment and is associated with long-term survival in well-differentiated pNETs, with reported 5-year overall survival generally >60–80% in localized disease. Everolimus improved median PFS from 4.6 to 11.0 months in RADIANT-3, and surufatinib improved median PFS from 3.7 to 10.9 months in SANET-p. PRRT (^177^Lu-DOTATATE) provides durable disease control, with median PFS commonly >20–30 months in well-differentiated GEP-NET/pNET series, and can occasionally enable conversion surgery in selected cases [17,57,63,65,66,68,69].

Overall, progress in pNETs management reflects a shift toward precision oncology. Molecular profiling, advanced imaging, refinement of grading systems, and integration of patient-reported outcomes are reshaping clinical decision-making. Continued research, standardized protocols, and expanded access to specialized care will be essential to further optimize outcomes for this diverse patient population.

## 10. Conclusions

Despite significant progress, pNETs remain a challenging and evolving area in oncology. Advances in surgery, systemic therapies, and diagnostics have improved patient outcomes, yet management continues to require a highly individualized approach. No single strategy is universally effective, and treatment must balance oncologic control with preservation of quality of life—particularly given the often-indolent clinical course of these tumors. Future progress will rely on a deeper understanding of tumor biology, the development of refined molecular profiling tools, and the integration of emerging therapeutic modalities into clinical practice. Addressing disparities in access to specialized care and promoting truly patient-centered management will also be essential. Ultimately, the evolution of pNET care will depend on the synergy between surgical innovation, therapeutic precision, and personalized care models, with the aim of achieving not only longer survival but also outcomes that are meaningful and sustainable across diverse clinical scenarios.

### Authors’ Final Practical Considerations on Diagnosis and Management

The diagnosis and management of pNETs require a multimodal strategy that incorporates imaging, biomarkers, EUS-FNA, and standardized grading systems (Table 2).

While CT and MRI remain essential for initial detection and staging, functional imaging—particularly ^68^Ga-DOTATATE PET—has become indispensable for early detection and more accurate staging. EUS-FNA remains central for diagnosis and grading, although variability in Ki-67 interpretation continues to pose challenges. Biomarkers can provide supportive information but should be viewed as complementary rather than definitive diagnostic tools. As the field advances, standardized protocols, improved training, and integration of novel technologies will be key to improving diagnostic accuracy and patient outcomes. Surgical management continues to evolve, with growing emphasis on minimally invasive techniques and individualized treatment strategies. Distal pancreatectomy is generally preferred when oncologic staging (including lymph node assessment) is needed or when the lesion is close to the main pancreatic duct, whereas **enucleation** remains appropriate for selected small, solitary insulinomas clearly away from the duct and without nodal suspicion, accepting a higher fistula risk in exchange for maximal parenchymal preservation. Although surgery retains a pivotal role, international guidelines and clinical studies highlight notable differences in managing specific scenarios, particularly in the presence of metastatic disease. In these cases, decisions are increasingly guided by tumor biology rather than solely by anatomical resectability. The systemic treatment landscape is rapidly expanding. The integration of molecular diagnostics with innovative targeted therapies, PRRT, and, in select cases, immunotherapy, holds promise for more personalized and effective management.

Therefore, an active surveillance strategy for asymptomatic, sporadic, non-functional, well-differentiated pNETs < 2 cm may be based on contrast-enhanced MRI every 6 months, with multiphasic CT reserved for patients in whom MRI is contraindicated and EUS used selectively for equivocal imaging findings and/or repeat tissue sampling. Surveillance should be planned for at least 5 years, with continued follow-up thereafter individualized according to stability and patient comorbidities. Triggers for surgery include sustained growth on serial imaging (pragmatically, >5 mm/year or >20% increase within 6–12 months), reaching or approaching 2 cm, onset of symptoms or hormonal hypersecretion, radiologic suspicion of nodal or distant disease, ductal obstruction, or evidence of grade progression on repeat biopsy when clinically actionable. Given the limited evidence on how to formally adjust surveillance in the presence of biopsy-related Ki-67 uncertainty, Ki-67 from EUS-guided sampling should be interpreted cautiously and integrated with radiologic behavior and clinical context; when results are borderline or discordant, repeat sampling and multidisciplinary discussion are advisable. Continued multicenter collaboration and translational research will be essential to refine these therapies and validate their long-term clinical impact.

Ultimately, harmonizing the major international guidelines into a single, pragmatic framework is essential to reduce current practice variability and enhance clinical applicability. This unified approach should standardize decision nodes for active surveillance versus resection in NF-pNETs < 2 cm, clarify the role and limitations of EUS-guided biopsy, including the reliability and interpretation of Ki-67–based grading on small samples, and define shared principles for sequencing SSA, targeted therapies, and PRRT in metastatic disease. Such convergence would support more consistent multidisciplinary tumor board decisions and promote more reproducible surgical and medical strategies across centers.

## Figures and Tables

**Table 1:** Practical approach to lymphadenectomy in pancreatic neuroendocrine tumors (pNETs).

Tumor Location/Procedure	Nodal Stations Typically Addressed	Suggested Minimum Node Yield	Enucleation: Is Lymphadenectomy Indicated?
Pancreatic head (pancreaticoduodenectomy)	Peripancreatic (anterior/posterior), pyloric, hepatoduodenal ligament, superior mesenteric/peri-SMA	≥8–12	Not applicable
Pancreatic body–tail (distal pancreatectomy)	Peripancreatic, splenic artery, splenic hilum	≥6–10	Not applicable
Central pancreatectomy	Peripancreatic ± splenic artery (as appropriate)	≥6	Not applicable
Enucleation (selected pNETs)	No systematic lymphadenectomy (regional nodes not routinely cleared)	Not defined	Not routinely; consider selective sampling only if suspicious nodes are identified pre-/intraoperatively

**Table 2:** Comparative overview of major international guidelines (ENETS, ESMO, NANETS, and Chinese societies) for the management of non-functional pancreatic neuroendocrine tumors (NF-pNETs).

Item	ENETS (Europe)	ESMO (Europe)	NANETS (North America)	China (CSNET/CMA and Related Guidance)
Active surveillance (non-functional pNET < 2 cm)	Active surveillance is an accepted option for selected asymptomatic ≤2 cm NF-pNETs, incorporating additional risk features beyond size (growth rate, ductal obstruction, suspicious nodes, grade if available).	Surveillance may be considered in selected small NF-pNETs; decisions are individualized based on growth kinetics, imaging risk features, and patient/surgical risk.	Clear size-based framing: <1 cm—observe; >2 cm—resect; 1–2 cm—individualize using clinical/imaging risk factors and grade (if available).	Historically more resection-leaning in some documents; more recent recommendations increasingly allow surveillance for low-risk/low-grade small tumors, but thresholds and adoption may vary by center and access to staging tools.
EUS-guided biopsy (EUS-FNA/FNB)	Useful when tissue will change management (surveillance vs. surgery; systemic therapy planning). Core biopsy (FNB) preferred when grading/biomarkers are required; acknowledge sampling limitations.	Recommended when confirmation and/or grading is needed; interpret results in context given intratumoral heterogeneity and potential discordance with surgical specimens.	Particularly helpful in the 1–2 cm “gray zone” to support surveillance vs. resection when feasible; limitations in representativeness are recognized.	Often emphasizes securing histology/grade when it affects treatment selection (especially in advanced/metastatic settings); practice may be more biopsy-forward where it drives access to therapies.
Ki-67 from EUS samples (role & limitations)	Ki-67 is central to grading but may be under- or overestimated on limited samples due to heterogeneity and counting variability; consider confirmatory sampling if “high-stakes” decisions hinge on Ki-67.	Same principle: Ki-67 guides prognosis/therapy but biopsy-based grading can be imperfect; integrate with imaging (SSTR/FDG when available), growth dynamics, and clinical course.	Ki-67 “when available” can inform decision-making (notably for 1–2 cm lesions), but its limitations on small samples are explicitly acknowledged.	Frequently stresses the need for Ki-67 assessment prior to systemic therapy decisions; some pathways prefer sampling a metastatic lesion when accessible to reflect dominant biology.
Therapy in metastatic disease (sequencing SSA/targeted/PRRT)	For well-differentiated, SSTR-positive disease: SSA often as backbone/early therapy in indolent or symptomatic disease; subsequent lines commonly include targeted therapy (e.g., everolimus, sunitinib/other TKIs depending on availability) and PRRT for SSTR-positive progressive disease. Sequencing is risk-adapted (tumor burden, tempo, SSTR/FDG phenotype, grade, symptoms).	Similar risk-adapted approach: SSA in appropriate SSTR-positive settings; targeted agents for progression; PRRT as a key option for SSTR-positive disease after progression and/or when clinically appropriate. Recognizes limited head-to-head data to mandate a universal sequence.	SSA for symptom control and antiproliferative benefit in appropriate patients; targeted therapy and/or PRRT selected based on SSTR expression, disease tempo, burden, and patient factors.	Algorithms typically include SSA, targeted therapies, chemotherapy (e.g., CAPTEM in some settings), and PRRT where available; sequencing is influenced by grade/biology and local access/approval pathways.

The table summarizes key areas of convergence and divergence regarding: (1) criteria for active surveillance in small (<2 cm) tumors, (2) indications and limitations of EUS-guided biopsy (FNA/FNB), (3) the role and interpretative caveats of Ki-67 assessment from biopsy samples, and (4) therapeutic sequencing in metastatic disease, including somatostatin analogues (SSA), targeted agents, chemotherapy, and PRRT. While all guidelines endorse risk-adapted, individualized decision-making integrating tumor size, grade, growth kinetics, imaging phenotype (SSTR/FDG), and patient factors, differences persist in size thresholds for surveillance, biopsy emphasis, and preferred treatment sequencing, often reflecting regional practice patterns, access to staging tools, and regulatory availability of therapies.

## Data Availability

The original contributions presented in this study are included in the article. Further inquiries can be directed to the corresponding author.
